# Oral Health Assessment Tool (OHAT) deputized to informal caregivers: Go or no go?

**DOI:** 10.1002/cre2.481

**Published:** 2021-08-30

**Authors:** Bach Van Ho, Liza J. M. van de Rijt, Roxane A. F. Weijenberg, Claar D. van der Maarel‐Wierink, Frank Lobbezoo

**Affiliations:** ^1^ Department of Orofacial Pain and Dysfunction Academic Centre for Dentistry Amsterdam (ACTA), University of Amsterdam and Vrije Universiteit Amsterdam Amsterdam The Netherlands; ^2^ Department of Oral Medicine Academic Centre for Dentistry Amsterdam (ACTA), University of Amsterdam and Vrije Universiteit Amsterdam Amsterdam The Netherlands

**Keywords:** community dwelling, informal caregivers, OHAT, older people, oral health

## Abstract

**Objective:**

Investigating the usability of the Dutch version of the Oral Health Assessment Tool (OHAT‐NL) with informal caregivers of community‐dwelling older people with suspected dementia, without specific training.

**Materials and methods:**

In accordance with guidelines for establishing the cultural equivalency of instruments, the OHAT was translated into Dutch. Fifteen informal caregivers of community‐dwelling older people with suspected dementia and, as a reference standard, a dentist assessed the oral health of the older people using the OHAT‐NL. The caregivers' scores were compared with the dentist's scores. The usability of the OHAT‐NL was rated on a 10‐point scale (0 = incomprehensible, and 10 = very user friendly) and investigated further through short structured interviews.

**Results:**

There were differences between the dentist's and caregivers' assessments of the individual categories of the OHAT‐NL. The specificity of the need to visit an oral health care professional was 100.0%, while the sensitivity was 78.6%. The informal caregivers concluded that the tool made them more aware of different aspects of oral health. The tool was rated with a mean score of 7.7 (SD 1.7).

**Conclusion:**

The OHAT‐NL could be a useful tool for informal caregivers without specific training to indicate whether the person they care for should visit an oral health care professional.

## 1 INTRODUCTION

The global population is aging substantially (Department of Economic and Social Affairs, 2002). Moreover, the number of community‐dwelling older people is also on the rise (Centraal Bureau voor de Statistiek, 2017; Roy et al., 2018), while, simultaneously, their general level of health is decreasing (World Health Organization, 2015). Furthermore, older people are maintaining their own dentition longer (Müller et al., 2007), at the same time as their daily oral hygiene care is diminishing, and their risk of developing oral health problems is increasing (Nazliel et al., 2012).

The oral health of older people with dementia is poor in comparison to older people without dementia (Delwel et al., 2017). Older people with dementia have an increased need for professional oral health care (Chalmers et al., 2003; Delwel et al., 2017), yet they are far less likely to visit dental practices regularly (Lee et al., 2015). In addition to this, older people with dementia tend to under‐report pain despite the presence of oral health problems (Delwel et al., 2018), due to a lack of verbal communication (Sampson et al., 2015). Given that pain is associated with the behavioral and psychiatric symptoms of dementia, identifying and treating such pain could help to reduce such symptoms (World Health Organization, 2003; Sampson et al., 2015). If informal caregivers had the ability to identify oral changes, or unhealthy oral situations, then they could seek proper professional oral health care on behalf of the older people they care for.

Screening tools for non‐oral health care professionals to assess oral health status have been developed previously (Chalmers et al., 2005). Indeed, the Brief Oral Health Status Examination (BOHSE) tool was specifically developed for older people with moderate to severe dementia (Kayser‐Jones et al., 1995). In 2005, the BOHSE was modified into the Oral Health Assessment Tool (OHAT), and subsequently validated for formal caregivers in residential care facilities (Chalmers et al., 2005).

The present study, conducted in The Netherlands, assessed the possibility of informal caregivers to evaluate the oral health status of the older people they care for through the use of the OHAT, without any specific training. Since this study was conducted in The Netherlands, the OHAT was formally translated into Dutch (Ho et al., 2019).

The aim of this study was to investigate the usability of the Dutch version of the Oral Health Assessment Tool (OHAT‐NL) for informal caregivers of community‐dwelling older people with suspected dementia, without specific training.

## 2 MATERIAL AND METHODS

To investigate the usability of the OHAT‐NL for informal caregivers of community‐dwelling older people with suspected dementia, participants from the “Don't Forget the Mouth!” (DFTM!) study (Ho et al., 2019) were asked to participate in the study. The study DFTM! is an oral health intervention study with the goal to improve the oral health of community‐dwelling frail older people in The Netherlands, by means of early recognition of decreased oral health status, decreased ability for daily oral hygiene self‐care, and the presence of oral health complaints as well as by establishing the need for interprofessional care (Ho et al., 2019).

The study was approved by the Medical Ethical Committee of the VU University Medical Centre (METc VUmc, with correspondence number: 2016.406).

### 2.1 Participants

For the purposes of this study, 15 informal caregivers of community‐dwelling older people with suspected dementia were asked to participate. There were no specific inclusion or exclusion criteria for the informal caregivers. The inclusion criteria for the older people were 65 years or older, community dwelling, and suspected of having dementia by their formal caregiver, viz., a district nurse. The exclusion criteria were a Mini Mental State Examination (MMSE) (Folstein et al., 1975) score of lower than 18, terminally ill, and intramural living. Written informed consent was obtained from all the participants.

### 2.2 Study design

The older people were visited at home by a dentist (one of us; Bach Van Ho) between June and October 2017. Both informal caregivers, and, as a reference standard, the dentist assessed the oral situation of the older people using the OHAT‐NL. The informal caregivers had no prior training and were thus using the OHAT‐NL for the first time. Both the caregivers and the dentist had access to gloves, a dental mirror (mirror holder: Carl Martin, 485CH chroom, Solingen, Germany; mirror: Intertek, front rhodium nr. 3, New York), and a dental probe (Intertek, CP12, New York). After the assessments, the dentist conducted a short, structured interview with each informal caregiver about the usability of the OHAT‐NL. In the event that the oral condition of the older person required further examination or dental treatment, the dentist advised a dental visit. If the older person did not have a dentist, one was recommended to them.

### 2.3 Demographic and descriptive data of the participants

The data from the DFTM! study that was collected via The Older People and Informal Caregiver Survey Minimum Data Set (TOPICS‐MDS) questionnaires (Lutomski et al., 2013; van den Brink et al., 2015) was obtained in order to assess the characteristics (age, gender, and relationship with older person) and informal care information of the informal caregivers, as well as the characteristics (age, gender, MMSE, and educational level), home care utilization, and dental information of the older people themselves. The dental information from the DFTM! study was supplemented by data from the clinical oral examination from the dentist (see above; Section 2.2).

### 2.4 Oral health assessment tool translated into Dutch

The OHAT assesses oral health in regards to eight different categories (i.e., lips, tongue, gums and tissues, saliva, natural teeth, dentures, oral cleanliness, and dental pain) (Chalmers et al., 2005). Each category is scored from 0 to 2; 0 = healthy situation, 1 = changes in the situation, and 2 = unhealthy situation. The total score of the eight different categories can vary from 0 to 16, with the lower scores suggesting a healthier oral situation. If someone has a score of 1 or higher, then they would be advised to visit an oral health care professional (Chalmers et al., 2005).

The OHAT was translated into Dutch using the forward‐backward approach, which is in accordance with the “Guidelines for establishing cultural equivalency of instruments” (Ohrbach et al., 2013). The forward translation from English into Dutch was conducted independently by two people fluent in English and Dutch (two of us; Bach Van Ho and Roxane A.F. Weijenberg, a dentist and a neurobiologist, respectively). The two forward translations were compared, and a consensus was reached via the aid of a third bilingual person (one of us; Frank Lobbezoo, a dentist and orofacial pain expert). The common forward translated version was then translated back into English by an external professional translator without any dental background, and compared to the original OHAT by all individuals involved in the translation procedure (Bach Van Ho, Roxane A.F. Weijenberg, Frank Lobbezoo, and the external professional translator). While there were a few discrepancies between the back‐translation and the original document, these were discussed and subsequently dropped, because the discrepancies were not of great significance and the translation was deemed to be adequate. On the basis of this discussion, consensus was therefore reached on the final Dutch version, the OHAT‐NL (Ho et al., 2019). The validity of the translation was confirmed by the level of congruence between the original version and the back‐translated English version.

### 2.5 Structured interviews

To assess the usability of the OHAT‐NL, the tool was rated on a 10‐point scale (0 = incomprehensible, and 10 = very user friendly), and investigated further through short, structured interviews with the informal caregivers. The interviews were conducted by the visiting dentist (one of us; Bach Van Ho). Four questions were asked, as shown in Table 1.

**Table 1:** Short, structured interview questions for informal caregivers, along with selective extracts that express contrasting opinions

Questions	Answers
1 What are your thoughts on looking at the mouth in this way? (Dutch: Wat vond u van deze manier om naar de mond te kijken?)	“It is great to look (at the mouth together) with you. It is notable to see how she eats, does she have enough (occlusal) surfaces?” “It is kind of intimate, (…) I have a better understanding of the oral status. I knew that some teeth were missing (…), but there are quite a few (teeth) gone, that is new.”
2 What was difficult for you on this form? (Dutch: Wat vond u moeilijk aan het formulier?)	“Saliva, where do you need to look at? Tongue, color that is fine, but there are cracks (on the tongue)…” “Chapped, dry…it could be not moist (the lips, what should I choose?). How should the gingiva look?…”
3 What was easy for you on this form? (Dutch: Wat vond u makkelijk aan het formulier?)	“It is easier to circle (individual words).” “The category gives context, but the rest is more difficult.”
4 How user friendly would you rate the form? Score: 1 = incomprehensible and 10 = very user friendly (Dutch: Hoe gebruiksvriendelijk vindt u het formulier? Cijfer: 1 = onbegrijpelijk en 10 = erg makkelijk in gebruik)	The mean of this answer was 7.7, with a range of 4.0–10.0, and a standard error of 1.7
5 Would you use the form again? (Dutch: Zou u het formulier nog een gebruiken?)	
a Why, would you/would you not? (Dutch: Waarom, wel/niet?)	“It is kind of useful if there are problems to (know) where you have to look at, where you need to pay attention to.” “No, belongs to care”
b In which situation would you like to use the form again? How frequently? (Dutch: In welke situatie zou u het formulier nog eens gebruiken? Hoe frequent?)	“I would ask the (health care professional of the) home care organization if there is something peculiar (in the mouth).” “For the health of my parents.”
Remarks	“More aware of what's going on (in the mouth).” “(…) It is difficult to fully write down what you see.”

### 2.6 Statistical analyses

Both the characteristics and informal care information about the informal caregivers, and the characteristics, home care utilization, and dental information about the older people were presented via descriptive statistics. The OHAT‐NL scores of the informal caregivers were compared to the reference standard, that is, the OHAT‐NL scores of the dentist. Both the scores for each category and the recommendation to visit an oral health care professional (i.e., an OHAT score of 1 or higher) were compared. A cross‐tabulation was performed for the agreement with true positives (TP), false positives (FP), false negatives (FN), and true negatives (TN). The specificity (SPC) and sensitivity (SEN) were calculated through the following formula: SPC = TN/(TN + FP) and SEN = TP/(TP + FN) (Mandrekar, 2010). If the level of agreement, specificity and sensitivity scores were below 50%, then this was considered as low, while above 80% for these respective criteria was considered good. All the statistical analyses were performed using IBM Statistics SPSS 26 (SPSS Inc., Chicago, Illinois).

The short, structured interviews were assessed following the non‐cross‐sectional analysis approach (Ritchie et al., 2003).

## 3 RESULTS

The characteristics and informal care information of the 15 informal caregivers is presented in Table 2. The mean age of the informal caregivers was 63.0 (SD 12.1); 86.7% were female; 66.7% was a daughter (−in‐law) or a son (−in‐law), and they spent, on average, 11.3 (SD 18.9) hours a week on caring. The mean score for the difficulty level of the care they provided for the person they care for (i.e., their loved one in Table 2) was 3.0 (SD 2.7), on a scale ranging from 0 to 10, where 0 represents “not difficult at all” and 10 represents “far too difficult.”The characteristics, home care utilization, and dental information of the 15 participating frail community‐dwelling older people are displayed in Table 3. The mean age of the participants was 84.7 (SD 9.8) years old; 46.7% were male; the mean MMSE was 24.9 (SD 3.7); while 53.3% of them visited a dentist or dental hygienist regularly.

**Table 2:** Characteristics and informal care information about the informal caregivers^a^

	N = 15
Age, mean (range, SD)	63.0 (47–85, 12.1)
Gender (%)	
Male	2 (13.3)
Female	13 (86.7)
Relationship with community‐dwelling older person (%)	
Husband/wife/life partner	4 (26.7)
Sister (−in‐law) or brother (−in‐law)	1 (6.7)
Daughter (−in‐law) or son (−in‐law)	10 (66.7)
Time spent caring for your loved one, mean (range, SD)	
Hours per week	11.3 (0.0–71.0, 18.9)
Difficulty level of taking care of the loved one^b^, mean (range, SD)	3.0 (0.0–8.0, 2.7)
Unknown (%)	2 (13.3)

^a^ Data of the Do not Forget The Mouth! (DFTM!) study (Ho et al., 2019) collected with The Older People and Informal Caregiver Survey Minimum Data Set (TOPICS‐MDS) questionnaires (Lutomski et al., 2013; van den Brink et al., 2015)

^b^ On a scale ranging from 0 to 10, where 0 represents “not difficult at all” and 10 represents “far too difficult.”

**Table 3:** Characteristics, home care utilization^a^, and dental information about the community‐dwelling older people^b^

	N = 15
Age, mean (range, SD)	84.7 (66.0–98, 9.8)
Gender (%)	
Male	7 (46.7)
Female	8 (53.3)
MMSE, mean (range, SD)	24.9 (18–29, 3.7)
Education (%)	
6 years of primary school, Iom school, mlk school (special education)	2 (13.3)
More than primary school/primary school without further completed education	4 (26.6)
Mulo/mms/mavo/secondary professional education	3 (20.0)
Hbs/gymnasium/atheneum (university entrance level)	2 (13.3)
University/tertiary education	4 (26.7)
Do you receive home care? For example, a community nurse, family care or home help (%)	
Yes	12 (80.0)
Hours per week, mean (range, SD)	6.9 (1.0–16.0, 5.0)
No	2 (13.3)
Unknown	1 (6.7)
Oral health status	
Oral status (%)	
Dentate without RDP	6 (40.0)
Dentate with maxillary RDP	4 (26.7)
Complete dental protheses	5 (33.3)
RDP maxillary fit^c^	
Good	3 (33.3)
Sufficiently	2 (22.2)
Average	2 (22.2)
RDP mandibular fit	
Good	1 (20.0)
Sufficiently	4 (80.0)
Dental Prosthesis Plaque (Augsburger and Elahi score), mean (range, SD)	2.8 (1.5–4.0, 1.1)
Number of teeth (dentate), mean (range, SD)	18.4 (7.0–26.0, 6.9)
Number of retained roots mean (range, SD)	0.5 (0.0–7.0, 1.8)
Dental Plaque (Sillness and Loe score), mean (range, SD)	0.9 (0.0–2.2, 0.7)
Do you sometimes go to the dentist/dental hygienist? (%)	
Yes, regularly	8 (53.3)
Yes, with problems	2 (13.3)
No, never	4 (26.7)
Other	1 (6.7)

Abbreviation: RDP, removable dental prosthesis.

^a^ Data of the Do not Forget The Mouth! (DFTM!) (Ho et al., 2019) study collected with The Older People and Informal Caregiver Survey Minimum Data Set (TOPICS‐MDS) questionnaires. (Lutomski et al., 2013; van den Brink et al., 2015).

^b^ Data from the DFTM! study.

^c^ Missing N = 2.

### 3.1 Oral health assessment tool (OHAT‐NL)

In Table 4, a comparison of the scores of the OHAT‐NL conducted by the informal caregivers and the dentist are presented in a cross‐tabulation. No person was suspected of having dental pain. The agreement (TP plus TN) varied from low to good, 60.0% for lips, 73.3% for the tongue, 53.3% for gums and tissue, 66.7% for saliva, 80.0% for natural teeth, 11.1% for dentures, 60.0% for oral cleanliness, and 100.0% for dental pain. The specificity of individual categories was moderate to good. The sensitivity of individual categories was moderate to good, except for the categories “gums and tissues,” “saliva,” and “dentures,” for which the sensitivity was low. The level of agreement on whether someone needed to visit an oral health care professional was 80.0%. The specificity and sensitivity scores for whether someone needed to visit an oral health care professional were 100.0 and 78.6%, respectively.

**Table 4:** Comparison of the scores on the Dutch Oral health assessment Tool (Ho et al., 2019) (OHAT‐NL) of the dentist and the informal carers

Category	N = 15	Score^d^	Informal caregiver (N)	Dentist (N)	OHAT‐NL score 1 or higher					
					TP (N) (%)	FP (N) (%)	FN (N) (%)	TN (N) (%)	SPc (%)	Sen (%)
Lips		0	8	12	2	5	1	7	58.3	66.7
	15	1	6	3	13.3	33.3	6.7	46.7		
		2	1	0						
Tongue		0	10	12	2	3	1	9	75.0	66.7
	15	1	5	3	13.3	20.0	6.7	60.0		
		2	0	0						
Gums and tissues		0	15	8	0	0	7	8	100.0	0.0
	15	1	0	7						
		2	0	0	0.0	0.0	46.7	53.3		
Saliva		0	13	8	2	0	5	8	100.0	28.6
	15	1	2	7						
		2	0	0	13.3	0.0	33.3	53.3		
Natural teeth		0	4	6	4	1	0	4	80.0	100.0
	10^a^	1	3	3						
		2	2	1	40.0	10.0	0.0	40.0		
		N/A	6	5						
Dentures		0	11	0	1	0	7	0	N/A	12.5
	9^b^	1	0	9						
		2	1	0	11.1	0.0	77.8	0.0		
		N/A	3	6						
Oral cleanliness		0	9	3	6	0	6	3	100.0	50.0
	15	1	6	10						
		2	0	2	40.0	0.0	40.0	20.0		
Dental pain		0	15	15	0	0	0	15	100.0	N/A
	15	1	0	0						
		2	0	0	0.0	0.0	0.0	100.0		
Visit dental professional (i.e., oral health care professional)^c^	15	0	4	1	11	0	3	1	100.0	78.6
		1 or higher	11	14	73.3	0.0	20.0	6.7		

Abbreviations: FN, false negative; FP, false positive; N/A, not applicable; Sen, sensitivity; SPc, specificity; TP, true positive; TN, true negative.

^a^ Informal caregiver wrote down not applicable natural teeth, while the dentist noticed natural teeth.

^b^ Informal caregiver scored for dentures, while the dentist did not notice the dentures.

^c^ A category score of 1 or higher indicates the need to visit an oral health care professional.

^d^ Scores elaborated by categories: Lips (0 = smooth, pink, moist; 1 = dry, chapped, or red at corners; 2 = swelling or lump, white/red/ulcerated patch; bleeding/ulcerated at corners); Tongue (0 = normal, moist, roughness, pink; 1 = patchy, fissured, red, coated; 2 = patch that is red and/or white, ulcerated, swollen); Gums and tissues (0 = pink, moist, smooth, no bleeding; 1 = dry, shiny, rough, red, swollen, one, ulcer/sore spot under dentures; 2 = swollen, bleeding, ulcers, white/red, patches, generalized redness, under dentures); Salvia (0 = moist tissues, watery and free flowing salvia; 1 = dry, sticky tissues, little saliva present, resident think they have a dry mouth; 2 = tissues parched and red, very little/no saliva present, saliva is thick, resident thinks they have a dry mouth); Natural teeth (0 = no decayed or broken teeth/roots; 1 = 1–3 decayed or broken teeth/roots or very worn down teeth; 2 = 4+ decayed or broken teeth/roots, or very worn down teeth, or less than 4 teeth); Dentures (0 = no broken areas or teeth, dentures regularly worn, and named; 1 = 1 broken area/tooth or dentures only worn for 1–2 h daily, or dentures not named, or loose; 2 = more than 1 broken area/tooth, denture missing or not worn, loose and needs denture adhesive, or not named); Oral cleanliness (0 = clean and no food particles or tartar in mouth or dentures; 1 = food particles/tartar/plaque in 1–2 areas of the mouth or on small area of dentures or halitosis (bad breath); 2 = food particles/tartar/plaque in most areas of the mouth or on most of dentures or severe halitosis [bad breath]); Dental pain (0 = no behavioral, verbal, or physical signs of dental pain; 1 = are verbal &/or behavioral signs of pain such as pulling at face, chewing lips, not eating, aggression; 2 = are physical pain signs [swelling of cheek or gum, broken teeth, ulcers], as well as verbal and/or behavior signs [pulling at face, not eating, aggression]) (Chalmers et al., 2005).

The results showed that the prevalence of the recommendation to visit an oral health care professional by the dentist was around 90%. With a sample size larger than 13 participants (Bujang & Adnan, 2016), a power of at least 89% was achieved with a lower bound confidence interval of 50% for a sensitivity of the OHAT‐NL of 90%, based on a target significance level of 5% (Bujang & Adnan, 2016).

### 3.2 Structured interviews

Table 1 presents selected extracts from the structured interviews with informal caregivers. Answers with contrasting opinions were purposefully chosen for inclusion. During the short, structured interviews, the informal caregivers described their use of the OHAT‐NL as interesting, special, and as being somewhat intimate. Only a few described the tool as being difficult. When asked specifically about these difficulties, the informal caregivers reported that it was difficult to choose between the options, and that it was not always clear which oral situation was normal or otherwise. In contrast, when asked about the easier aspects of the tool, they singled out the descriptions of the categories as being helpful for scoring oral health. They rated the tool with a mean score (range, SD) of 7.7 (4.0–10.0, 1.7) on a scale from 0 to 10, where 0 represents incomprehensible, and 10 very user friendly. Caregivers from a younger generation (daughter [−in‐law{s}] or son [−in‐law{s}]) rated the tool higher in terms of being user friendly than caregivers who were from the same generation (partners, sister [−in‐law], or brother [−in‐law]) as the older people. When asked if they would use the tool again, several caregivers reported they would if the person they care for indicated problems or asked them to take a look, while others stated that they would prefer a health care professional to use the tool and examine further. At the end of the interview, the informal caregivers concluded that the use of the OHAT‐NL made them more aware of the different aspects of oral health. Some even indicated that the tool could be expanded upon, so that they would have the opportunity to write down more information about the person they cared for.

## 4 DISCUSSION

The aim of this study was to investigate the usability of the OHAT‐NL for informal caregivers of community‐dwelling older people with suspected dementia.

A recent article (Marchini et al., 2019) concluded that the oral health of older people is negatively impacted by cognitive decline. Moreover, oral health problems can go unnoticed, leading to pain, which, in turn, can negatively impact upon behavior (Marchini et al., 2019). Involving informal caregivers in oral health assessment, specifically via the OHAT‐NL, can contribute both to the recognition of oral health problems and oral pain, and to people seeking proper professional oral health care.

Some of the informal caregivers indicated that they would use the OHAT‐NL again, while others indicated that they would prefer a health care professional to use the tool or make an assessment. Daughter (−in‐law[s]) or son (−in‐law[s]) reported higher rates than partners, sister (−in‐law), or brother (−in‐law) for the OHAT‐NL. This can be explained by the nature of the relationship they have with those they care for, or the different generational attitudes towards and expectations of health care professionals (Devoe et al., 2009). Nevertheless, the informal caregivers concluded that the use of the OHAT‐NL made them more aware of the different aspects of oral health. However, the informal caregivers did describe some difficulties with scoring when it was not clear which oral situation was normal or otherwise. The use and completion of the OHAT has been studied previously (Chalmers et al., 2005) through the use of focus‐group questionnaires with carers in a residential care facility. The carers reported that it was difficult to understand some of the categories, and that they would like a video or written materials as a reference point when using the OHAT.

Furthermore, there was variation in the level of agreement for the individual categories of the OHAT‐NL between the informal caregivers and the dentist. In previous oral health assessment studies that either used the OHAT or the comparable revised oral assessment guide (ROAG) (Andersson et al., 2002; Chalmers et al., 2005; Klotz et al., 2019), categories such as teeth, dentures, saliva, and dental pain were similarly deemed difficult to assess. In the current study, the category “dentures” yielded a low level of agreement. This could be explained by the fact that the lack of a name on dentures was usually overlooked by informal caregivers, as it is not relevant for community‐dwelling older people. Hence, removal of this description under the category “dentures” should be considered. Moreover, the level of agreement for other categories was moderate, with the exception of the categories “natural teeth” and “dental pain,” which displayed a good level of agreement. The specificity of individual categories was moderate to good, thus indicating that informal caregivers were able to identify a healthy oral situation. The sensitivity was moderate to good, with the exception of the individual categories “gums and tissues,” “saliva,” and “dentures.” For these categories, the sensitivity score was low, thus indicating that it was challenging for informal caregivers to identify oral changes or an unhealthy oral situation. Of course, it was expected that there would be some categories with a low sensitivity score, as a result of the lack of training in how to use the OHAT‐NL (Chalmers et al., 2005; Klotz et al., 2019). Despite this, the recommendation to visit an oral health care professional was found to have a good level of agreement, specificity, and sensitivity. Therefore, even without specific training, the OHAT‐NL could prove to be a useful tool for identifying the need to consult an oral health care professional.

### 4.1 Strengths and limitations

The strength of this study derives from the involvement of informal caregivers in the assessment of the oral health status of those they care for using the OHAT‐NL, without any prior training, which to the best of our knowledge has hitherto not been done. While this study investigated the use of the OHAT‐NL in relation to a range of oral situations, there was no dental pain discovered during the course of this study, which can be seen as a limitation. Further research is thus needed in this respect to assess the level of specificity, sensitivity, and agreement. Moreover, there were no substantive changes in content made during the process of translating the OHAT‐NL. Given this lack of methodological or conceptual changes, the structure of assessing in one language could thus be compared to the structure of assessing in another language (Ohrbach et al., 2013). This means that validity of the OHAT‐NL could be confirmed by looking at the level of agreement between the original version and back‐translated English version of the OHAT. Furthermore, in the analysis, the scores of each category were collapsed between 0 and 1 with 2. In other words, the sensitivity and sensitivity of a normal (score 0) and abnormal (scores 1 and 2) situation was assessed. Although this showed whether a visit to an oral health care professional was recommended, it did not reflect the specificity and sensitivity of each given score. Finally, this study had a small sample size. Therefore, any conclusions are tentative, and further research is recommended.

### 4.2 Implications

The OHAT‐NL was used without specific training by the informal caregivers, with good results on the need to visit an oral health care professional. The quick application makes the OHAT‐NL more accessible and feasible for everyday usage. Use of the OHAT‐NL by informal caregivers could thus have the added value of maintaining the oral health of community‐dwelling older people with suspected dementia. Furthermore, the OHAT‐NL could increase awareness about the different aspects of oral health among informal caregivers.

## 5 CONCLUSION

The OHAT‐NL could be a useful tool for informal caregivers, as it requires no specific training and allows them to identify whether the person they care for, that is, the community‐dwelling older person with suspected dementia, should visit an oral health care professional.

### 5.1 Recommendations

Further research on oral health in community‐dwelling older people with dementia, and the involvement of informal caregivers is strongly recommended. A follow‐up to this study with a minimum sample size of 34 participants is recommended (Bujang & Adnan, 2016). This minimum sample size is recommended to detect as many true‐positives as possible with the OHAT‐NL, and to narrow the range of the confidence interval in such a way that the lower bound of the confidence interval is 70% instead of 50% (Bujang & Adnan, 2016). Furthermore, another follow‐up to this study could involve a comparison between informal caregivers and formal caregivers. A qualitative study with informal caregivers could shed more insight into their knowledge of oral health, experiences with daily oral health care, the barriers to dental visits for the person they care for, and the amount of time they spend caring. Such a qualitative study could further elaborate upon the usability of the OHAT‐NL with informal caregivers.

## CONFLICT OF INTERESTS

The authors declare that they have no competing interests with respect to the research, authorship, and/or publication of this article.

## AUTHOR CONTRIBUTIONS

Bach Van Ho and Roxane A.F. Weijenberg conceived and designed the study. Bach Van Ho planned and conducted the study, and wrote the manuscript. Liza J.M. van de Rijt was a major contributor in writing the manuscript. Claar D. van der Maarel‐Wierink obtained the funding and contributed in writing the manuscript. Frank Lobbezoo took part in obtaining funding and revising the manuscript. All authors read and approved the final manuscript.

## Data Availability

The data that support the findings of this study are available from the corresponding author upon reasonable request.
